# FERM Domain Containing Protein 7 Interacts with the Rho GDP Dissociation Inhibitor and Specifically Activates Rac1 Signaling

**DOI:** 10.1371/journal.pone.0073108

**Published:** 2013-08-13

**Authors:** Jiali Pu, Yanfang Mao, Xiaoguang Lei, Yaping Yan, Xiaoxiong Lu, Jun Tian, Xinzhen Yin, Guohua Zhao, Baorong Zhang

**Affiliations:** Department of Neurology, Second Affiliated Hospital, College of Medicine, Zhejiang University, Hangzhou, Zhejiang, China; University of Birmingham, United Kingdom

## Abstract

The FERM domain containing protein 7 gene (*FRMD7*) associated with the X-linked disorder idiopathic congenital nystagmus (ICN) is involved in the regulation of neurite elongation during neuronal development. Members of the Rho family of small G-proteins (Rho GTPases) are key regulators of the actin cytoskeleton and are implicated in the control of neuronal morphology. The Rho GDP dissociation inhibitor alpha, RhoGDIα, the main regulator of Rho GTPases, can form a complex with the GDP-bound form of Rho GTPases and inhibit their activation. Here, we demonstrate that the full length of the mouse FRMD7, rather than the N-terminus or the C-terminus alone, directly interacts with RhoGDIα and specifically initiates Rac1 signaling in mouse neuroblastoma cell line (neuro-2a). Moreover, we show that wild-type human FRMD7 protein is able to activate Rac1 signaling by interacting with RhoGDIα and releasing Rac1 from Rac1-RhoGDIα complex. However, two missense mutations (c.781C>G and c.886G>C) of human FRMD7 proteins weaken the ability to interact with RhoGDIα and release less Rac1, that induce the activation of Rac1 to a lesser degree; while an additional mutant, c.1003C>T, which results in a C-terminal truncated protein, almost fails to interact with RhoGDIα and to activate Rac1 signaling. Collectively, these results suggest that FRMD7 interacts with one of the Rho GTPase regulators, RhoGDIα, and activates the Rho subfamily member Rac1, which regulates reorganization of actin filaments and controls neuronal outgrowth. We predict that human mutant FRMD7 thus influences Rac1 signaling activation, which can lead to abnormal neuronal outgrowth and cause the X-linked ICN.

## Introduction

Mutations in the gene encoding the FERM domain containing protein 7 (*FRMD7*; NM_194277) are associated with the X-linked idiopathic congenital nystagmus (ICN) [1–5]. FRMD7 downregulation has been shown to alter the development of neurites by influencing the dynamics of F-actin during retinoic acid (RA)-induced differentiation in mouse neuroblastoma (Neuro-2a) cells [6]. However, the precise mechanism by which FRMD7 influences F-actin, and its involvement in ICN pathogenesis, remains to be elucidated.

The human *FRMD7* gene comprises 12 exons, encodes a 714-residue polypeptide, and shares a four-point-one, ezrin, radixin, moesin (FERM) domain at its N-terminus. The FERM family of proteins regulates the adhesion and morphogenesis of cells by modulating changes in the cytoskeleton [7,8], where the FRMD7 protein is thought to be involved in signal transduction between the plasma membrane and cytoskeleton. Furthermore, FRMD7 shares close amino acid sequence homology with two other FERM domain containing proteins: FARP1 (FERM, RhoGEF and pleckstrin domain protein 1; chondrocyte-derived ezrin-like protein; NM_005766) and FARP2 (FERM, RhoGEF and pleckstrin domain protein 2; NM_014808). FARP1 and FARP2 play important roles in neuronal development through activating Rho GTPase signaling. FARP1 is known to promote the dendritic growth of spinal motor neuron subtypes by activating RhoA signaling [9,10], while FARP2 has been shown to modulate the length and degree of branching of neurites in developing cortical neurons via the Rac1 signaling pathway [8,11].

Rho GTPases are key regulators of the actin cytoskeleton in eukaryotic cells and mediate morphological changes during neuronal development and plasticity, such as the growth of neurites, axon guidance and dendrite elaboration [12–15]. RhoA, Cdc42 and Rac1 form the archetypal trio of Rho GTPases whose function as signaling switches resides in their ability to cycle between active GTP-bound states and inactive GDP-bound states.

Rho GTPases have three regulators. GTPase-activating proteins (GAPs) stimulate the intrinsic rate of GTP hydrolysis, inactivating GTPases [16,17]. Guanine nucleotide exchange factors (GEFs) promote the exchange of GDP for GTP and directly activate Rho GTPases [18]. The Rho GDP dissociation inhibitor (GDI) forms a complex with the GDP-bound inactive form of Rho GTPases and inhibits their activation [19]. This last complex is not activated by the GDP/GTP exchange factor for Rho family members, suggesting the presence of another factor necessary for this activation.

Interestingly, FARP1 and FARP2 both function as GEFs [8,11]. Furthermore, ERM proteins directly interact with RhoGDI and initiate the activation of Rho small G-proteins [20]. In our previous work, we identified two novel missense mutations and a truncated mutation of human *FRMD7* in three X-linked ICN pedigrees [3]. Therefore, in this study we investigated the role and mechanism of FRMD7 regulation of neuronal cytoskeletal dynamics through the Rho GTPases signaling pathway and the related pathogenesis of mutant FRMD7 leading to the X-linked ICN.

## Materials and Methods

### Experimental animals

The mice used in this study were purchased from the Animal Center, School of Medicine, Zhejiang University (Hangzhou, Zhejiang, China). All experimental procedures were approved by the Institutional Committee at Zhejiang University.

### RNA isolation and reverse transcription PCR

Total RNA was isolated from embryonic 18-day (ED18) mouse brains and HEK293T cells using TRIZOL reagent (Invitrogen, San Diego, CA) according to the manufacturer’s instructions. 5 µg RNA was reverse transcribed using oligo dT by reverse transcriptase. For PCR ampliﬁcation, speciﬁc oligonucleotide primer pairs (10 pmol each) (Table 1) were incubated with 2.5 µL cDNA template in 25 µL PCR reaction mixtures containing 1.5 mM MgSO_4_, mixed deoxynucleotides (1 mM each), and 0.5 U KOD FX PLUS (Toyobo, Japan) polymerase. Dilutions of the cDNAs were amplified for 30~35 cycles at 94° C for 2 min, 98° C for 10 s, 60° C for 40 s, and 68° C for 120 s. The amplified PCR products were analyzed by 1.5% agarose gel electrophoresis and ethidium bromide staining.

**Table 1:** Primers for amplification of the mouse FRMD7 and Rho GTPases related genes.

Name	Sense primers (5'–3')	Antisense primers (5'–3')	Product length (bp)
Full-FRMD7	TGGATCCATGCTCCATTTAAAAGTG	GCTCGAGTTAAGCTAAGAAATAATTGC	2112
Nr-ferm FRMD7	TGGATCCATGCTCCATTTAAAAGTG	CCGCTCGAGCCTGAAGAAAGCATGGTA	837
Cr-fragment FRMD7	CCGCTCTCCGAAGAGCCCAAATCAAA	GCTCGAGTTAAGCTAAGAAATAATTGC	1275
Rho GDIɑ	TCGGATCCATTATGGCAGAACAGGAACC	TCTCGAGTCAGTCCTTCCACTCCTTT	615
RhoA	GTATGGATCCGTTATGGCTGCCATCAGG	GAATCTCGAGCAAGATGAGGCACCCAG	582
Rac1	GGGATCCCCATGCAGGCCATCAAGTG	CCTCGAGCAACAGCAGGCATTTTCTC	579
Cdc42	GTTTGGATCCCCATGCAGACAATTAAGTG	CTTGCTCGAGTAGCAGCACACACC	573

### Plasmid construction

Each PCR product was confirmed by subcloning into the pGEM-T Easy vector (Promega, Madison, WI, USA) and sequencing. Mouse and human full-length FRMD7 cDNA, as well as a 837 bp fragment (Nr-ferm) encoding the FERM domain of FRMD7 (amino acids (aa) 1–279) and a 1275 bp fragment (Cr-fragment) encoding the △FERM domain of FRMD7 (aa 280–703), were FLAG-tagged at the C-terminus and digested with BamHI and XhoI, before subcloning into pcDNA3.1(+) vector (Invitrogen). Mutations of human FRMD7 were constructed by overlapping PCR. Mouse or human RhoGDIɑ cDNA was also digested with BamHI and XhoI, and subcloned into pCMV-N-Myc vector (Beyotime, Jiangsu, China). HA-tagged Rac1, Cdc42 and RhoA were subcloned into pcDNA3.1(+) vector after digestion with BamHI and XhoI. For prokaryotic expression, the constructs for wild-type mouse RhoGDIɑ, Rac1/Cdc42-binding domain of mouse PAK2 (aa 66–147) and RhoA-binding domain of Rhotekin (RBD) in PGEX-5X-1 to produce glutathione S-transferase (GST) fusion proteins were made as previously described [21].

### Expression and purification of recombinant proteins

Bacterially expressed recombinant RhoGDIɑ, PAK2 and RBD proteins were puriﬁed as previously described [22]. *Escherichia* strain BL21(DE3) transformed with the plasmids was treated for 4 hr at 37° C with 1 mM isopropyl-thio-D-galactoside (IPTG) to induce the expression of each protein, which was then puriﬁed through a glutathione-Sepharose 4B column.

### Cell cultures and transient transfections

Mouse neuroblastoma (Neuro-2a) cell line and HEK293T cell line were purchased from the Chinese Academy of Sciences Committee Type Culture Collection Cell Bank/CAS Shanghai Institutes for Biological Sciences Cell Resource Center (Shanghai, China) and mouse embryonic fibroblast (NIH3T3) cell line was purchased from KeyGEN Biotech (Nanjing, Jiangsu, China). Neuro-2a cells, HEK 293T and NIH3T3 cells were cultured in Dulbecco’s modified Eagle’s medium (DMEM; Invitrogen, Carlsbad, CA, USA) containing 10% fetal bovine serum (FBS; Invitrogen), penicillin and streptomycin. Cultures were maintained in 5% CO_2_ at 37° C, and were passaged every 2–3 days. Transient transfections were carried out using Attractene Transfection Reagent (Qiagen, Valencia, CA, USA) according to the manufacturer’s protocol.

### Co-immunoprecipitation of FRMD7 and RhoGDIɑ

Flag-tagged mouse full length FRMD7 and Myc-tagged RhoGDIɑ were co-transfected into Neuro-2a cells using Attractene Transfection Reagent (Qiagen, Valencia, CA, USA). Co-immunoprecipitation was performed 48 h after the transfection. Cells were lysed on ice for 30 min with lysis buffer (50mM Tris (pH 7.4), 150mM NaCl, 1% NP-40, 0.25% sodium deoxycholate, sodium orthovanadate, sodium fluoride, EDTA, leupeptin) with 1% cocktail (Sigma, St. Louis, Missouri). The lysates were centrifuged at 13,000 g for 25 min at 4° C and the supernatants were incubated with the anti-Flag antibody (Sigma, St. Louis, Missouri) or anti-Myc antibody (Abmart, Shanghai, China) at 4° C overnight. The immunocomplex was then collected with protein A/G Sepharose (Santa Cruz Biotechnology, Santa Cruz, California) for 2 h at 4° C. The pellets were washed four times with lysis buffer (without cocktail) and diluted by sample loading buffer, then boiled for 5 min. This was followed by immunoblot analysis using anti-Myc antibody (Abmart, Shanghai, China) or anti-Flag antibody (Sigma, St. Louis, Missouri).

### Immunoprecipitation assay

For the immunoprecipitation assay, transient expression of Myc-RhoGDIɑ with or without Flag-full-length FRMD7 was carried out using pCMV-Myc-RhoGDIɑ and pcDNA3.1(+)-Flag-full-length FRMD7 in Neuro-2a cells or HEK293T cells. Myc-RhoGDIɑ was precipitated with 3 µg of anti-Myc monoclonal antibody bound to 50 µl of protein A/G-Sepharose, followed by centrifugation and extensive washing with lysis buffer. Comparable amounts of the pellets were subjected to SDS-polyacrylamide gel electrophoresis, and the separated proteins were electrophoretically transferred to a nitrocellulose membrane sheet. The sheet was processed using the ECL detection kit (Pharmacia Biotech Inc.) to detect Myc-RhoGDIɑ, Rac1, CDC42, RhoA and Flag-FRMD7 with the anti-Myc (Abmart, Shanghai, China), anti-Rac1 (Millipore, Boston, USA), anti-Cdc42 (Cell signaling, Boston, USA), anti-RhoA (Cell signaling, Boston, USA) and anti-Flag monoclonal antibodies (Sigma, St. Louis, Missouri) as primary antibodies, respectively.

### Pull down GTPase assay

*In vivo* GTPase assay was performed according to the protocol of ProFound Pull-Down GST Protein: Protein Interaction Kit (Thermo number 21516). HA-tagged Rac1, RhoA and Cdc42 were respectively co-transfected into Neuro-2a cells with Flag-tagged FRMD7 using Attractene Transfection Reagent (Qiagen, Valencia, CA, USA), cultured for 48 h, and lysed (50mM Tris (pH 7.4), 150mM NaCl, 1% NP-40, 0.25% sodium deoxycholate, sodium orthovanadate, sodium fluoride, EDTA, leupeptin). Cell lysates were clariﬁed by centrifugation, and the supernatant was incubated with 100 µg of GST-PAK2 protein immobilized on glutathione-Sepharose beads for 3 min. Beads were washed with washing buffer (20mM H EPES, pH 7.4, 142.5mM NaC l, 1% Nonidet P-40, 10% glycerol, 4mM EGTA, and 4mM EDTA), and bound GTP-Rho proteins were detected by Western blotting with the anti-HA monoclonal antibody (Abmart, Shanghai, China). *In vivo* RhoA assay was performed according to the method reported by Schwartz et al using a GST fusion to the RhoA-binding domain of Rhotekin (GST-RBD) [23]. In human wild-type and mutant-type FRMD7 Rac1 and Cdc42 GTPase assay, HA-tagged human Rac1 or Cdc42 was co-transfected into HEK293T cells with Flag-tagged wild-type or mutant-type FRMD7 using Attractene Transfection Reagent (Qiagen, Valencia, CA, USA), cultured for 48 h, lysed and precipitated by GST-PAK2 protein.

### Immunofluorescence and F-actin staining

For immunocytochemistry, NIH3T3 cells were grown on chamber slides to 50–60% conﬂuence in 24-well plates, then transfected with 0.4 µg plasmid DNA and 1.5 µL Attractene Transfection Reagent (Qiagen, Valencia, CA, USA) per well. After 24 h, the medium was replaced with DMEM without serum for 16 h. Cells ﬁxed in 4% formaldehyde in PBS for 15 min at room temperature were stained. After three washes in PBS, cell nuclei were stained with 4', 6-diamidino-2-phenylindole (DAPI; ZhongShan Goldenbridge Biotechnology Company Ltd, Beijing, China, 1:5000) for 5 min at room temperature. F-actin was stained using TRITC-conjugated rhodamine–phalloidin (77481, Sigma Aldrich, St Louis, MO, USA) fluorescein diluted in PBS and bovine serum albumin for 45 min at room temperature. Morphological features were quantiﬁed using a confocal laser scanning microscope (Leica TCS SP5 X, Wetzlar, Germany).

### Statistical analysis

All values are expressed as the mean ± standard error of the mean. The differences between the two groups were compared using unpaired t-tests. A difference of p < 0.05 was considered significant.

## Results

### Mouse full-length FRMD7 associated with RhoGDIα

**Figure 1:**
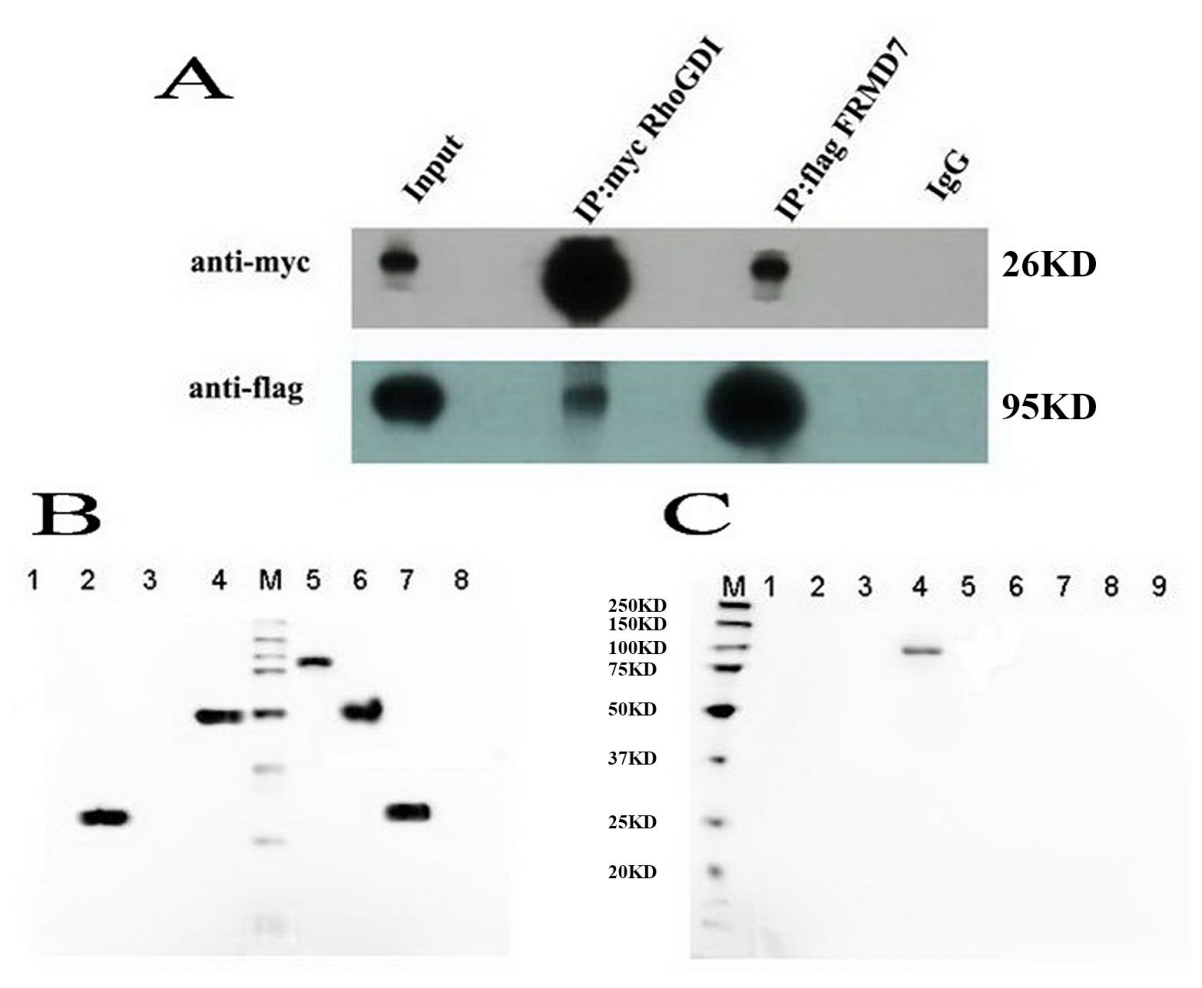
Mouse full-length FRMD7 directly interacts with RhoGDIα in vivo and in vitro. We co-transfected mouse pCMV-Myc-RhoGDIα and full-length pcDNA3.1(+)-Flag FRMD7 into Neuro-2a cells. (A) Reciprocal co-immunoprecipitation experiments showed that pcDNA3.1(+)-Flag FRMD7 co-immunoprecipitates with pCMV-Myc-RhoGDIα, and vice versa, in Neuro-2a cells. Normal mouse IgG antibody served as the negative control. (B) The prokaryotic expression of GST-RhoGDIα in DE3, and eukaryotic expression of full-length FRMD7 and truncates, Lane 1: PGEX-5X-1, -IPTG; Lane 2: PGEX-5X-1, +IPTG; Lane 3: PGEX-5X-1-RhoGDIα, -IPTG; Lane 4: PGEX-5X-1-RhoGDIα, +IPTG; M: Marker; Lane 5: FRMD7-FL; Lane 6: Cr-FRMD7; Lane 7: Nr-FRMD7; Lane 8: Empty vector. (C) *In vitro* GST pull down experiments, using highly purified GST-RhoGDIα recombinant proteins, where full-length FRMD7, but not Nr-ferm (aa: 1-279; the N-terminal FERM domain of the full-length FRMD7) or Cr-fragment (aa: 280-703; truncated FERM domain of FRMD7), was found to directly interact with RhoGDIα. M: marker; Lane 1: GST-RhoGDIα; Lane 2: GST-RhoGDIα+Cr-FRMD7; Lane 3: GST-RhoGDIα+Nr-FRMD7; Lane 4: GST-RhoGDIα+FRMD7-FL; Lane 5: GST- RhoGDIα+Empty vector; Lane 6: Cr-FRMD7; Lane 7: Nr-FRMD7; Lane 8: FRMD7-FL; Lane 9: Empty vector.

We first examined whether the full-length FRMD7 is associated with RhoGDIα. Neuro-2a cells were co-transfected with FLAG-tagged FRMD7 and Myc-tagged RhoGDIα. In the anti-FLAG precipitates, the anti-Myc antibody revealed the interaction between FRMD7 and RhoGDIα. In the anti-Myc precipitates, the anti-FLAG antibody also detected the association of FRMD7 with RhoGDIα (Figure 1a).

### Direct interaction between FRMD7 and RhoGDIɑ

We then examined the direct physical interaction of RhoGDIɑ with FRMD7 using highly purified GST recombinant proteins. RhoGDIɑ was found to directly interact with full-length FRMD7, but not Nr-ferm (aa: 1-279; the N-terminal FERM domain of the full-length FRMD7) or Cr-fragment (aa: 280-703; truncated FERM domain of FRMD7), suggesting that direct interaction of RhoGDIα with FRMD7 requires both the FERM domain and the C-terminal end, which plays a key role in subcellular localization [24,25] (Figure 1b).

### FRMD7 activates Rac1 signaling

**Figure 2:**
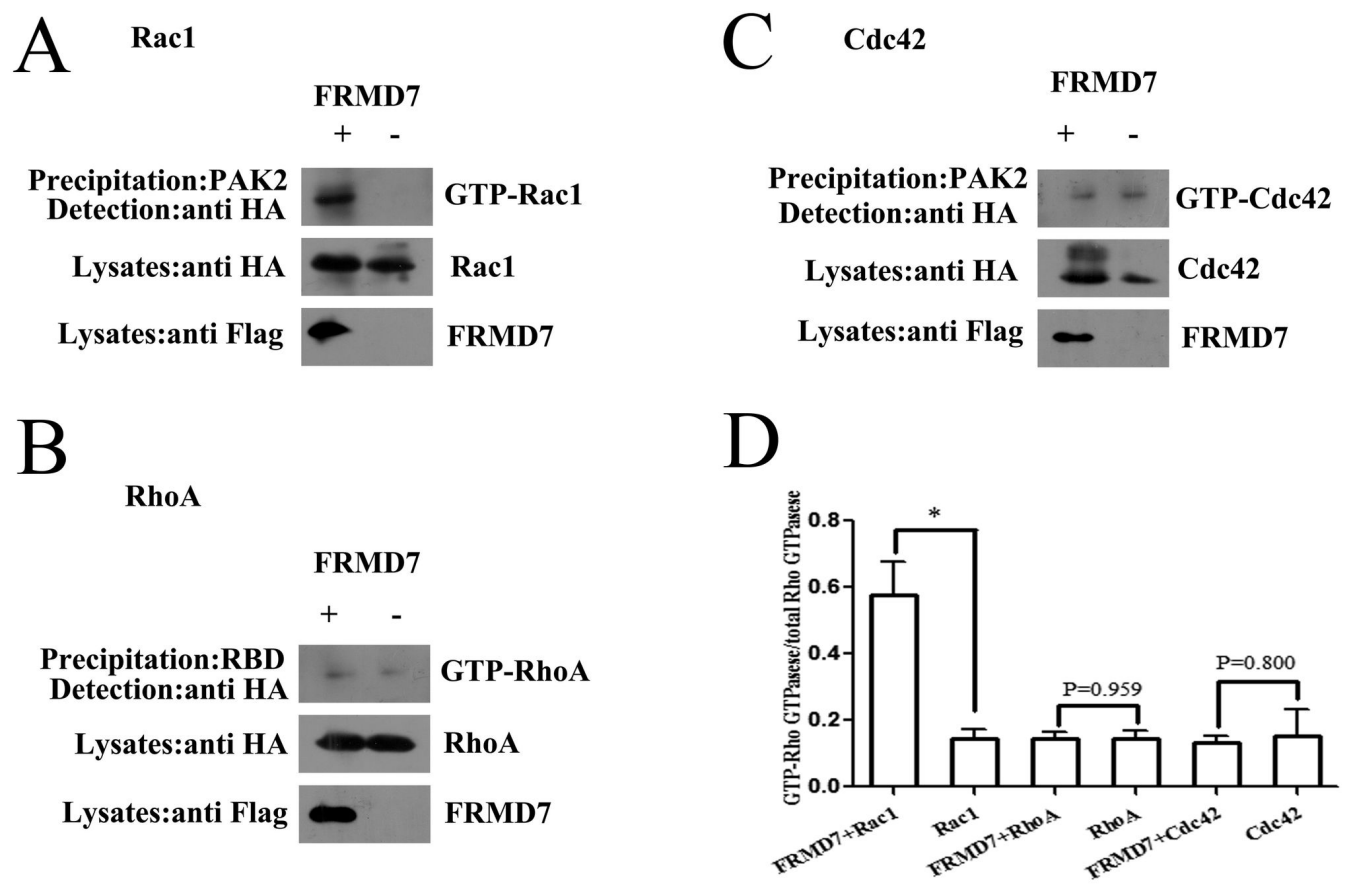
FRMD7 specifically activates Rac1 but not RhoA or Cdc42 signaling. HA-tagged Rac1, RhoA and Cdc42 were respectively co-transfected into Neuro-2a cells with FLAG-tagged FRMD7, cultured for 48 hours, and lysed. Cell lysates were clariﬁed by centrifugation, and the supernatant was incubated with GST-PAK2 protein (GTP-Rac1/Cdc42 binding domain) immobilized on glutathione-sepharose beads, where bound GTP-Rho proteins were detected by Western blotting with anti-HA monoclonal antibody. RhoA activity was examined using GST fusion to the RhoA-binding domain of Rhotekin (GST-RBD). Extracts of Neuro-2a cells transfected with Rac1 and FRMD7 contained increased amounts of GTP-Rac1 compared with the control cells (Figure 2A). Conversely, little or no activation of RhoA or Cdc42 was detected (Figure 2B and 2C). The experiments were repeated five times, where the graphs (Figure 2D) represent the average of five independent experiments (columns, mean; bars, S.E.M.; *p < 0.05, **p < 0.01).

To determine whether FRMD7 can speciﬁcally catalyze GDP/GTP exchange, we measured the activity of Rac1, RhoA and Cdc42 in Neuro-2a cells transiently transfected with or without FRMD7 by afﬁnity precipitation. Because Rho GTPases in the GTP-bound state bind to their downstream effectors, GST fusions of these effectors can be used to capture active Rho GTPases from cell lysates. Thus, a GST fusion to the Rac1/Cdc42 binding domain of PAK2 (GST-PAK2) was used to speciﬁcally precipitate GTP-bound Rac1 or Cdc42 from cell extracts [21]. RhoA activity was examined using a GST fusion to the RhoA-binding domain of Rhotekin (GST-RBD) [23]. This assay revealed that extracts of Neuro-2a cells transfected with Rac1 and FRMD7 contained increased amounts of GTP-Rac1 compared with the control cells (Figure 2A). Conversely, little or no activation of RhoA or Cdc42 was detected (Figure 2B and 2C).

### FRMD7 releases Rac1 from its complex with RhoGDIα

**Figure 3:**
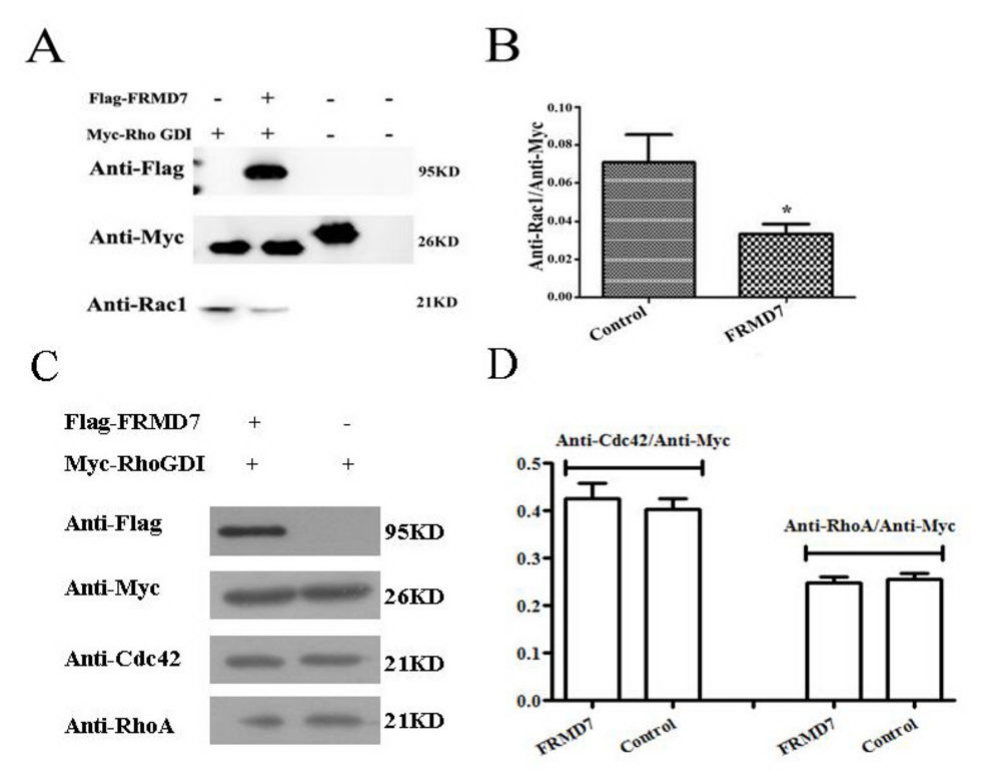
FRMD7 releases Rac1 from its complex with RhoGDIα. Endogenous Rho GTPases were co-immunoprecipitated with Myc-tagged RhoGDIα (Myc-RhoGDIα) from the lysate of Neuro-2a cells transiently expressing Myc-RhoGDIα alone. However, in the lysate of cells transiently expressing both Myc-RhoGDIα and FLAG-tagged FRMD7, less endogenous Rac1 was co-immunoprecipitated with Myc-RhoGDIα, while FLAG-tagged FRMD7 was co-immunoprecipitated with Myc-RhoGDIα (Figure 3A). The level of Cdc42 and RhoA remain similarly via the co-immunoprecipitated with Myc-RhoGDIα (p > 0.05) (Figure 3C). Neuro-2a cells transfected with Myc-RhoGDIα alone and normal Neuro-2a cells served as negative controls. The Myc-tag fusion protein acted as a positive control. The experiments were repeated five times, where the graphs (Figure 3B, 3D) represent the average of five independent experiments (columns, mean; bars, S.E.M.; *p < 0.05, **p < 0.01).

*In vivo*, RhoGDIα forms a complex with Rho GTPases and inhibits the activation of Rho GTPases by GEFs. Given the fact that FRMD7 can activate Rac1 signaling, this raises the possibility that FRMD7 reduces the activity of RhoGDIα, thus facilitating the release of Rac1 from RhoGDIα. We next examined whether FRMD7 regulates RhoGDIα activity in intact cells. Endogenous Rho GTPases were co-immunoprecipitated with Myc-tagged RhoGDIα (Myc-RhoGDIα) from the lysate of Neuro-2a cells transiently expressing Myc-RhoGDIα alone, suggesting that endogenous Rho GTPases were complexed with exogenous RhoGDI in intact cells (Figure 3). However, in the lysate of cells transiently expressing both Myc-RhoGDIα and FLAG-tagged FRMD7, less endogenous Rac1 was co-immunoprecipitated with Myc-RhoGDIα (p < 0.05), but FLAG-tagged FRMD7 was co-immunoprecipitated with Myc-RhoGDIα (Figure 3A, 3B), suggesting that in the RhoGDIα complex Rac1 was replaced or partly replaced by FLAG-tagged FRMD7 to form RhoGDI-FLAG-tagged FRMD7 complexes in Neuro-2a cells. The level of Cdc42 and RhoA remain similarly via the co-immunoprecipitated with Myc-RhoGDIα (p > 0.05). These results show that FRMD7 can directly interact with RhoGDIα and reduce its ability to inhibit the GDP/GTP exchange reactions of Rac1.

### Effects of FRMD7 on the actin cytoskeleton

**Figure 4:**
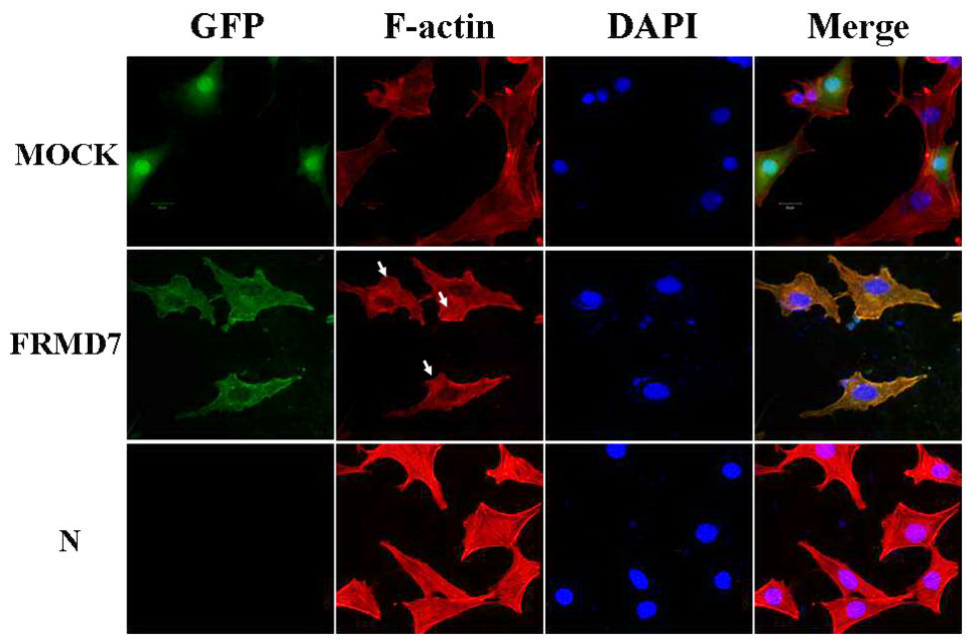
Effect of overexpression of FRMD7 on the actin cytoskeleton in NIH3T3 cells. NIH3T3 cells transfected with mouse FRMD7-pEGFP-n1 (green) or the empty pEGFP-n1 vector (green) (Mock) were cultured for 24 hours and then cultured for 16 hours without FBS. F-actin was stained with TRITC-conjugated rhodamine-phalloidin (red). NIH3T3 cells transfected with the mouse full-length FRMD7 displayed some lamellipodia and ruffles, consistent with Rac1 activation. Normal NIH3T3 cells served as the control (N). Scale bars: 20 µm.

We further examined whether overexpression of FRMD7 mimics the functions of Rho family members. Rac1, RhoA and Cdc42 each elicit distinct morphological changes. Speciﬁcally, Rac1 induces lamellipodia formation and membrane rufﬂing, RhoA induces stress ﬁber formation and Cdc42 induces filopodia extension [26,27]. We transfected a mouse embryonic fibroblast cell line (NIH3T3) with the plasmid for GFP-fused FRMD7 or GFP. After serum starvation for 16 hours, we detected the expression of FRMD7 by GFP autofluorescence and examined the actin structures by staining F-actin with rhodamine-phalloidin. In GFP-FRMD7-transfected cells, some lamellipodia and ruffles were detected, consistent with Rac1 activation [27,28] (Figure 4). Stress ﬁber formation was signiﬁcantly suppressed in the cells overexpressing FRMD7 compared with the control cells expressing GFP.

### Mutations of human FRMD7 influence the activation of Rac1 signaling

**Figure 5:**
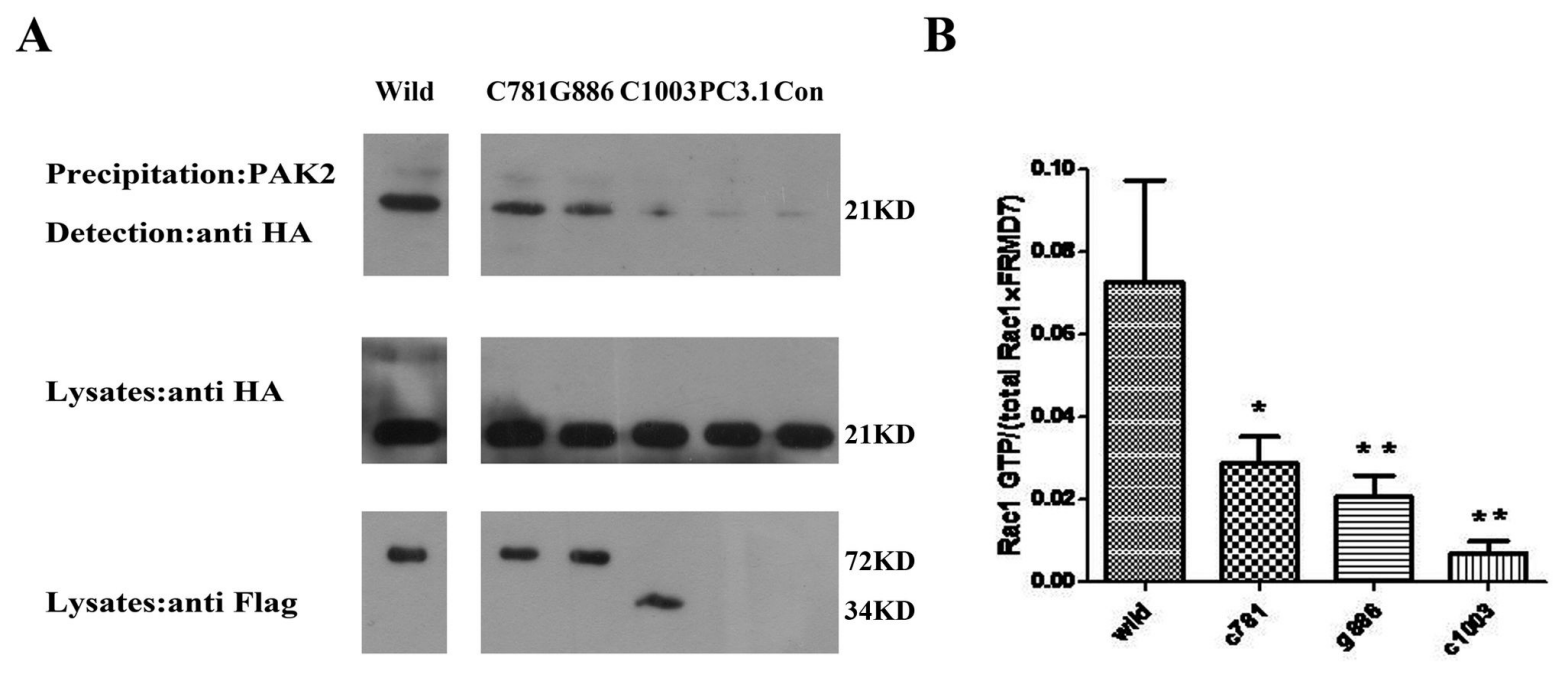
Effect of human mutant-type FRMD7 proteins on the activation of Rac1 signaling. HA-tagged human Rac1 was co-transfected into HEK293T cells with Flag-tagged wild-type (Wild) or mutant-type (c.781C>G, c.886G>C and c.1003C>T) human FRMD7, cultured for 48 hours, and lysed. Cell lysates were clariﬁed by centrifugation, and the supernatant was incubated with GST-PAK2 protein immobilized on glutathione-sepharose beads, where bound GTP-Rac1 proteins were detected by Western blotting with anti-HA monoclonal antibody. Extracts of HEK293T cells transfected with two missense mutant-type FRMD7 (c.781C>G, C781, c.886G>C, G886) showed decreased amounts of GTP-Rac1 compared with the wild-type FRMD7. Little activation of Rac1 was detected in extracts of HEK293T cells transfected with another truncated mutant-type FRMD7 (c.1003C>T, C1003). PC3.1: pcDNA3.1+Rac1; CON: Rac1 only. The experiments were repeated five times, where the graphs represent the average of five independent experiments (columns, mean; bars, S.E.M.; *p < 0.05, **p < 0.01).

To investigate whether mutations in human FRMD7 influence Rac1 signaling compared with the wild-type, we assessed the amount of activated Rac1 in human HEK 293T cells transiently co-transfected with human wild-type or mutant-type FRMD7 and Rac1 by afﬁnity GST-PAK2 precipitation [21]. We found that the human wild-type FRMD7 protein had the ability to activate Rac1 signaling, however two missense mutant FRMD7 (c.781C>G and c.886G>C) proteins previously reported by us generated less GTP-Rac1 compared with the wild-type (P < 0.05). Furthermore, little activation of Rac1 was detected in another truncated mutant-type group, c.1003C>T, (P < 0.01) which leads to a C-terminal truncated protein and changes in the normal subcellular localization pattern [3,24,25] (Figure 5).

### Mutations of human FRMD7 reduce the ability to interact with RhoGDIα and release less Rac1

**Figure 6:**
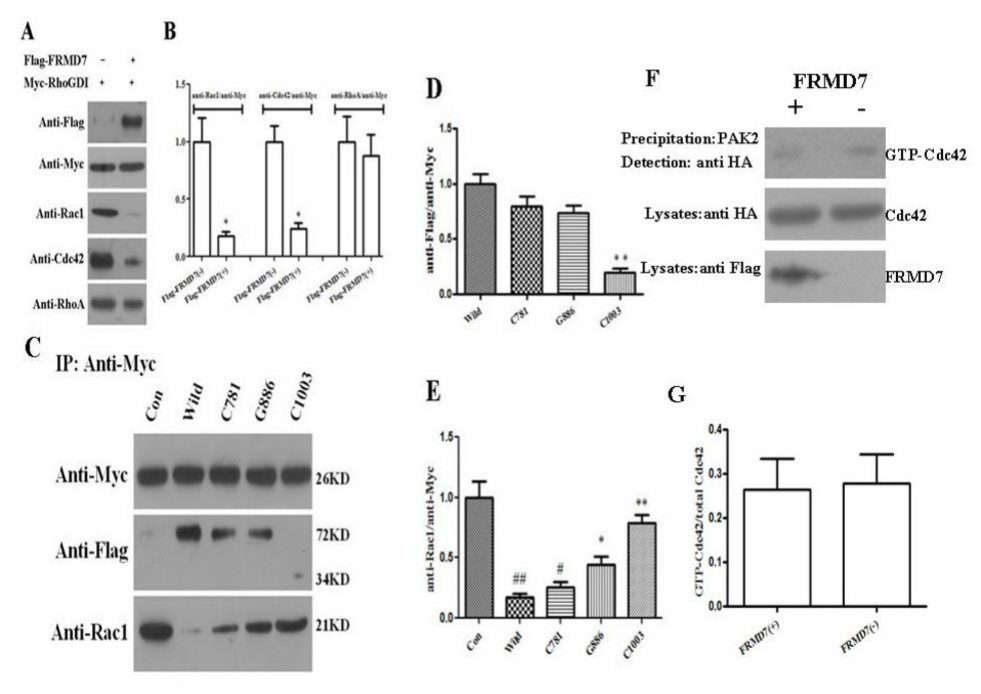
Influence of human wild and mutant-type FRMD7 proteins on RhoGDIα and its activity. HEK293T cells were transfected with Myc-tagged RhoGDIα alone or together with FLAG-tagged wild-type or mutant-type FRMD7. Anti-Flag, endogenous Rac1, Cdc42 and RhoA were co-immunoprecipitated with Myc-tagged RhoGDIα (Myc-RhoGDIα) from the lysate of HEK293T cells transfected after 48h. In the lysate of cells transiently expressing both Myc-RhoGDIα and FLAG-tagged FRMD7, less endogenous Rac1 and Cdc42 were co-immunoprecipitated with Myc-RhoGDIα, and RhoA remained unchanged, while FLAG-tagged FRMD7 was co-immunoprecipitated with Myc-RhoGDIα (Figure 6A and 6B). Furthermore, Extract of HEK293T cells transfected with two missense mutant-type FRMD7 (c.781C>G, C781, c.886G>C, G886) showed decreased amounts of FLAG-tagged FRMD7 and increased level of Rac1 compared with the wild-type one after anti-Myc immunoprecipitated. For another truncated mutant-type FRMD7 (c.1003C>T, C1003), little amount of FRMD7 was detected in the extracts immunoprecipitated by anti-Myc, therefore, almost equal amount of Rac1 was observed compared to the control one (Figure 6C, 6D and 6E). HEK293T cells transfected with Myc-RhoGDIα alone served as negative controls. In addition, we have tested whether human FRMD7 activated Cdc42 by using GST-PAK2 protein precipitation. The results showed the human wild-type FRMD7 protein could not activate Rac1 signaling (Figure 6F and 6G). The experiments were repeated five times, where the graphs (Figures 6B, 6D, 6E and 6G) showed the statistical results (columns, mean; bars, S.E.M.; *p < 0.05 versus Flag-FRMD7 (-) in Figure 6B; *p < 0.05, **p < 0.01 versus wild type; ^#^ p < 0.05, ^# #^ p < 0.01 versus Con in Figure 6 D and 6E).

We further examined whether mutations in human FRMD7 also influence the association with RhoGDIα and its activity. HEK293T cells were transfected with Myc-tagged RhoGDIα alone or together with FLAG-tagged FRMD7. After the anti-Myc precipitates, the anti-FLAG antibody also detected the association of FRMD7 with RhoGDIα, and less endogenous Rac1 was co-immunoprecipitated with Myc-RhoGDIα compared to the condition of expressing Myc-RhoGDIα alone. Surprisingly, Cdc42 has given arise to the similar phenomenon with Rac1, and the co-immunoprecipitated amount of another Rho GTPase RhoA was the same (Figure 6A and B). Furthermore, mutant FRMD7 proteins reduced the ability to interact with RhoGDIα and released less Rac1 from Rac1-RhoGDIα complex. Especially for the truncated mutant-type, c.1003C>T, almost failed to interact with RhoGDIα and release Rac1 (Figure 6C, D and E). In addition, we have tested whether human FRMD7 activated Cdc42 by using GST-PAK2 protein precipitation. The results showed the human wild-type FRMD7 protein could not activate Rac1 signaling.

## Discussion

The *FRMD7* gene is a member of the super 4.1 family of proteins, sharing a four-point-one, ezrin, radixin, moesin (FERM) domain at its N-terminus. In a previous study, a significant reduction in the overall length of neurites was detected using RA-induced neuronal differentiation, when *FRMD7* was knocked down in Neuro-2a cells by RNA interference [6]. However, the precise biochemical role of FRMD7 in neuronal development and neurite extension remains poorly understood.

Our data reveals an association of FRMD7 with Rho-GDIα dependent on both the N-terminus (the FERM domain) and the COOH-terminus of FRMD7, the latter playing a key role in subcellular localization [24]. Previous studies have shown that ERM proteins can interact with RhoGDI, thereby inhibiting its function and promoting the activation of Rho small G-proteins [20]. ERM proteins contain at least two functionally different domains, the N-terminal FERM domain and the C-terminal domain [29–31]. Interestingly, full length ERM proteins have been found to fold in a manner such that the N- and C-terminal regions mask each other, preventing them from interacting with RhoGDI and actin filaments, respectively [32–34]. Only the N-terminus fragment of ERM proteins can associate with RhoGDI and activate Rho GTPases signaling, but only full-length FRMD7 will enable this to happen. FRMD7 contains at least three domains: the N-terminus, the FA region and the C-terminus. In our study, we tested the N-terminal FERM domain and a truncated version, but neither interacted with RhoGDIα. This suggests that the mechanism by which FRMD7 exerts its function is different from that of ERM proteins, or possibly that the FA domain is also involved, but this requires further investigation. Meanwhile, it may give some explanation as to why mutations present in different domains eventually lead to the same disease.

Our study demonstrated that FRMD7 has the ability to release Rac1 from RhoGDIα *in vitro*, where activation of Rac1 signaling through FRMD7 may be attributable. The release of Rho from RhoGDIα is an important step allowing the GDP-bound form of Rho to be activated by guanine nucleotide exchange factors (GEFs) and to become associated with the membrane. The RhoGDIα displacement factor, ezrin/radixin/moesin, also from the FERM family of proteins, induces activation of RhoA in Swiss 3T3 cells [20]. The neurotrophin receptor p75NTR involved in the regulation of axonal elongation can also activate RhoA [35]. Thus, in a similar manner to these proteins, FRMD7 might appear to act as a displacement factor to activate Rac1 signaling, but this needs further investigation.

Furthermore, we demonstrated that two missense mutant-type (c.781C>G and c.886G>C) human FRMD7 proteins, which lead to an arginine in the protein 261 loci being substituted for glycine (p.R261G) and glycine in the protein 296 loci being substituted for arginine (p.G296R) respectively, reduced the ability to associate with RhoGDIα, released less Rac1 from Rac1-RhoGDIα complex and activated less Rac1. Another mutant-type, c.1003C>T, which results in the arginine of the protein 335 loci to be substituted for a stop codon (p.R335X) and leads to a COOH-terminal truncated protein, exhibits a nuclear localization pattern and does not co-localize with the cytoplasmic distribution of F-actin, showed little ability to interact with RhoGDIα, release Rac1 and activate Rac1 signaling [3,24]. As the Rac1 signaling pathway appears to be involved in the regulation of neurite extension in the developmental stage, mutations of the *FRMD7* gene which alter the regulation of Rac1 signaling may be linked to the pathogenesis of idiopathic congenital nystagmus.

Recent research suggests RhoGDIα may be involved in the spatial and temporal activation of the downstream Rac1 signaling pathway [36]. Future studies should explore whether the spatial control of Rac1 signaling by FRMD7, as regulated by RhoGDIα, has an impact on neuronal development.

Three mammalian RhoGDIs have been identified: the ubiquitously expressed RhoGDI1 (or RhoGDIα) [37,38], hematopoietic cell-selective RhoGDI2L (or y/D4GDI) [39,40] and RhoGDI3 which is expressed in the lungs, brain and testes [41,42]. RhoGDIs were initially characterized as simply Rho GTPase inhibitors; however, their function is now known to be more complex, with a central regulatory role in Rho GTPase activation [36]. However, each of the three mammalian RhoGDIs interacts with multiple Rho GTPases [43], making it difficult to establish how a single Rho GTPase can be selectively released from RhoGDIs and activated, suggesting that other proteins such as GDI dissociation factors (GDF) may play precise roles in the regulation of RhoGDIα and Rho GTPases.

Here, we have shown that human FRMD7 releases both Rac1 and Cdc42 from RhoGDIα, however only Rac1 can be activated. Recently, Etienne et al [43] describes a novel effect of RhoGDIα to stabilize Rho GTPases. The limited amount of RhoGDIα in cells generates a competitive balance between Rho GTPases in order to prevent their degradation. They also show the activity of released Rho GTPases from RhoGDIα was either elevated or unchanged. To some degree, it is similar to our result of both Rac1 and Cdc42 can be released from RhoGDIα but only Rac1 is activated. In addition, there are some references, demonstrating other GDF, such as ERM (ezrin, radixin, moesin) proteins [20], the neurotropin receptor (p^75NTR^) [35] and the tyrosine kinase Etk [44]. In those studies, they only test whether the activated Rho GTPase can be released from RhoGDIα, but did not test whether the inactive Rho GTPases. Therefore, so far, we do not know exactly these GDF selectively displace specific Rho GTPase. Furthermore, the exact role of RhoGDIα in the Rho GTPases cycle remains unclear. Why the released Cdc42 is not been activated and did they degradation or play other roles in the pathway? These questions should be illustrated in future.

In summary, FRMD7 interacts with RhoGDIα, and specifically activates Rac1 signaling, which is involved in neuronal development. Mutations of human FRMD7 alter the regulation of Rac1 signaling, which might be a potential mechanism behind the pathogenesis of XL-ICN.
